# Reduced-cost microwave modeling using constrained domains and dimensionality reduction

**DOI:** 10.1038/s41598-023-45890-x

**Published:** 2023-10-28

**Authors:** Slawomir Koziel, Anna Pietrenko-Dabrowska, Ubaid Ullah

**Affiliations:** 1 Engineering Optimization and Modeling Center, Reykjavik University, 102 Reykjavík, Iceland; 2 Faculty of Electronics, Telecommunications and Informatics, Gdansk University of Technology, 80-233 Gdańsk, Poland; 3 Networks and Communication Engineering Department, Al Ain University, P.O. Box 112612, Abu Dhabi, United Arab Emirates

**Keywords:** Electrical and electronic engineering, Computational science

## Abstract

Development of modern microwave devices largely exploits full-wave electromagnetic (EM) simulations. Yet, simulation-driven design may be problematic due to the incurred CPU expenses. Addressing the high-cost issues stimulated the development of surrogate modeling methods. Among them, data-driven techniques seem to be the most widespread owing to their flexibility and accessibility. Nonetheless, applicability of approximation-based modeling for real-world microwave components is hindered by a high nonlinearity of the system characteristics, dimensionality issues, and broad ranges of operating parameters the model should cover to make it practically useful. Performance-driven modeling frameworks deliver a partial mitigation of these problems through appropriate spatial orientation of the metamodel domain, which only encapsulates high-quality designs and not the entire space. Unfortunately, the initial model setup cost is high, as defining the domain requires database designs that need to be a priori acquired. This paper introduces a novel approach, where the database designs are replaced by random observables, and dimensionality of the domain is reduced using spectral analysis thereof. The major contributions of the work include implementation of the explicit dimensionality reduction of the confined surrogate model domain and introducing this concept into a complete cost-efficient framework for modeling of microwave components. Comprehensive benchmarking demonstrates excellent performance of the introduced framework, both in terms of predictive power of the rendered surrogates, their scalability properties, as well as low computational overhead associated with the model setup.

## Introduction

Geometrical complexity of microwave passive components and devices has been recently growing to meet the demands of various application areas (space communications^1^, mobile communications^2^, automotive radars^3^, internet of things, IoT^4^, microwave imaging^5^, energy harvesting^6^, wireless power transfer^7^, etc.), and to provide the required functionalities (e.g., multi-band operation^8^, harmonic suppression^9^, reconfigurability^10^). Accurate evaluation of circuits is instrumental in their design processes, yet, analytical and equivalent network models, traditionally used for this purpose, are no longer reliable in many cases. This is especially true for compact structures featuring strong cross-coupling effects, which is a result of tightly arranged layouts rendered by miniaturization techniques such as folding of transmission line^11^, or employment of slow-wave phenomenon (e.g., compact microwave resonant cells, CMRCs^12^). Full-wave electromagnetic (EM) simulations provide the only versatile means that can be used for reliable evaluation of arbitrary circuit geometries, also at the existence of environmental components (nearby devices or connectors), while accounting for the coupling effects, dielectric and radiation losses, substrate anisotropy, and others.

EM simulations have been omnipresent in the design of microwave components, yet their utilization for solving tasks that require multiple circuit evaluations may be hindered by the incurred computational expenses. This might be a limiting factor even when local search is concerned (e.g., gradient-based procedures^13^), whereas it is particularly troublesome for global optimization^14,15^, uncertainty quantification, UQ (e.g., statistical analysis^16^, yield maximization^17^), or multi-criterial design^18,19^. Many of these tasks require hundreds and thousands of system evaluations when solved using conventional methods, such as Monte Carlo simulation in statistical design^20^, or direct EM-driven global optimization using bio-inspired population-based algorithms^21,22^. There have been plenty of methods developed to expedite simulation-based design procedures, including simplistic approaches, e.g., supervised parametric studies^23,24^ (still widely used in practice), or worst-case analysis for UQ^25^. Still, rigorous numerical methods have also gained an increased popularity, especially in the context of local search (adjoint sensitivities^26^, sparse gradient updating techniques^27–29^, mesh deformation^30^, parallelization^31^). Nevertheless, the class of procedures that have been attracting particular attention in the recent years are surrogate-based algorithms^32–35^. These can be categorized as physics-based^36^, and data-driven^37^. Physics-based methods (space mapping^38–40^, shape-preserving response prediction^41^, adaptive response scaling^42^, manifold mapping^43^) rely on an underlying low fidelity model, which, for microwave components, is typically an equivalent network^44^, but it can also be coarse-discretization EM model^30^. The problem-specific knowledge encoded therein permits constructing reliable surrogates using only a small number of EM-evaluated samples. Still, physics-based models are not as flexible as data-driven ones^45^, thus they are more frequently used for local rather than global search. Data-driven surrogates are significantly more popular as they are easy to handle, versatility, and a plethora of third-party implementations (e.g.,^46,47^) available free of charge. Among widely used techniques kriging^48^, neural networks^49–51^, radial basis functions^52^, support vector machines^53^, Gaussian process regression (GPR)^54^, ensemble learning^55^, polynomial chaos expansion (PCE)^56^ may be listed. Surrogate-based design frameworks often incorporate machine learning schemes^57^ and sequential sampling^58^, either to diminish the cost of acquiring training data or to enable global search capabilities under more challenging scenarios^59^. Other acceleration methods that do not directly fall into any of the two aforementioned categories, are feature-based optimization (FBO)^60^, along with cognition-driven design^61^, exploiting a particular shape of the component’s outputs (e.g., allocation of resonances^62^, local pass-band maxima^63^, etc.). Therein, reformulating the design task in terms of suitably defined characteristic (feature) points, allows for reducing the nonlinearity of the associated objective functions, thereby leading to faster convergence of the optimization procedures.

Replacing EM simulations by fast surrogates to facilitate design procedures that require massive system evaluations is the main rationale behind surrogate-assisted methods^64^. However, the curse of dimensionality and high nonlinearity of microwave component outputs hinder a construction of stand-alone surrogates that would be valid over broad ranges of system parameters and its operating conditions. Availability of the latter could potentially simplify EM-driven design procedures by eliminating the need for iterative prediction-correction schemes pertinent to most optimization frameworks that rely on surrogate models^65–67^. Instead, the employment of conventional algorithms would be sufficient. Due to the issues mentioned above, library-like reusable metamodels can only be built for simple components characterized by a small number of parameters, often in tight ranges of these^68,69^. At this point, one should mention available mitigation methods, e.g., orthogonal model pursuit^70^, or high-dimensional model representation (HDMR)^71^. Still, these techniques are not multipurpose ones. On the other hand, variable-fidelity methods (two-stage GPR,^73^, co-kriging^72^, Bayesian model fusion^74^]) are more versatile at the expense of implementation complexity.

The recent performance-driven (or constrained) modeling methods^75–79^ present a conceptually different approach to alleviating the difficulties pertaining to approximation techniques. The focus is on a proper definition of the metamodel domain, which is restricted to the regions accommodating designs of superior quality with regard to the intended design targets. The volume of such a domain is to a large extent smaller than that of the conventional design space (typically, an interval delimited by the lower and upper variable bounds), which permits a rendition of accurate models at low computational cost. The preeminent performance-driven technique is the nested kriging framework^76^, and its enhancements using variable-fidelity models^80^, and dimensionality reduction^81^. At the same time, as constrained modeling methods rely on pre-optimized reference design sets to identify the regions of interest, the initial model setup cost may be high (at least a few hundreds of EM simulations). This may be somewhat mitigated by utilization of sensitivity data^82^, which leads to a reduction of the required number of reference designs. In^83^, an advanced constrained modeling approach has been proposed, where the reference designs are replaced by random observables, and the model domain is defined using information extracted therefrom. As demonstrated, this leads to a significant reduction of the initial costs without being detrimental to the model predictive power.

This paper introduces a novel technique that belong to performance-driven modeling frameworks. It employs the concepts presented in^83^ while bringing in additional advances, specifically a reduction of the domain dimensionality. The latter is carried out by means of the principal component analysis of the observable data, and spanning the domain along the most important eigenvectors of the covariance matrix corresponding to this data. The modeling framework implemented based on these concepts retains the overall benefits of the reference-design-free confined modeling, including the low setup cost, while enabling enhanced scalability (with respect to the cardinality of training data set), and even further improved modeling accuracy. Numerical experiments conducted for three microwave components (compact couplers and a power divider) fully demonstrate these advantages, as well as corroborate that the proposed technique outperforms both conventional modeling methods and the previously reported performance-driven frameworks. Furthermore, suitability of surrogate models constructed using our method for design purposes is illustrated by means of a variety of application studies involving parameter tuning of the considered circuits under different design scenarios. The novel contributions of the work include: (i) explicit dimensionality reduction of the confined surrogate model domain using the principal components of the observable set, (ii) implementation of a complete cost-efficient framework for modeling of microwave components, (iii) demonstrating the ability of setting up reliable models valid over a wide range of geometric parameters and operating conditions using small training data sets, (iv) demonstrating accuracy and scalability improvements enabled by dimensionality reduction, (v) demonstrating superior performance of the proposed technique in terms of accuracy of the constructed surrogates and computational efficacy over several state-of-the-art benchmark techniques.

## Reference-design-free domain-confined modeling with dimensionality reduction

This section introduces the proposed approach to modeling of microwave components. Its fundamental components include domain confinement realized by means of information garnered from a collection of random observables spread over the design space, as well as a reduction of the domain dimensionality realized using the principal component analysis of the observable set. The section is organized as follows. The reference-design-free constrained modeling approach is recalled in “Reference-Design-Free Domain-Confined Modeling” Section. A definition of a reduced-dimensionality domain is provided in “Reduced-Dimensionality Model Domain. Construction of Final Surrogate” Section, whereas the complete modeling framework is summarized in “Proposed Modeling Procedure: Complete Workflow” Section.

## Reference-design-free domain-confined modeling

The modeling approach introduced in this paper falls into the category of performance-driven techniques^75^. The fundamental concepts of this paradigm are outlined in brief in this section for the convenience of the reader. The vast majority of data-driven modeling methods focus on the best possible exploration of available data^65^, as well as improvements concerning design of experiments^46^, e.g., to allocate additional points in the regions corresponding to higher nonlinearity of the circuit outputs. However, from the perspective of design utility of the model, most of the traditionally defined parameter space, i.e., an interval demarked by lower and upper bounds on the system variables, comprises inferior-quality designs. Performance-driven modeling attempts to identify the subsets containing high-quality designs and only construct the surrogate model therein. The designs that are superior from the viewpoint any given set of performance requirements (e.g., required return loss levels, power split, phase relations, etc., over target operating frequencies or bandwidths) normally occupy low-dimensionality manifolds, the volume of which is tiny in comparison to the original (interval-like) design space. This leads to the following benefits^75^:

- The metamodel can be established with the use of a considerably smaller number of data samples, in contrast to conventional domains;
- The curse of dimensionality can be overcome to a large extent;
- Domain confinement does not limit neither the ranges of the design variables nor the operational conditions the surrogate is valid within.

The notation has been explained in Table 1^75^. Among the listed items, the main principle adopted by performance-driven modeling is the space *F* of design requirements. Specifically, the validity region of the metamodel is determined with respect to *F* rather than the design space *X*. This is because our goal is to construct the model that—for the sake of its design utility—adequately represents the system responses over the required ranges of performance figures. This puts the modeling process in a different perspective, in which the particular subset of the parameter space to become the domain of the model is secondary with respect to the objective space.

**Table 1:** Basic notation utilized in domain-confined modeling.

Design objectives		Circuit parameters	
*f*_*k*_, *k* = 1, …, *N*	Performance figures encoding design objectives (e.g., operating frequency/frequencies, bandwidth, substrate permittivity)	*x*_*i*_, *i* = 1, …, *n*	Parameter of the circuit under design (typically, independent dimensions that undergo refinement)
*F* *f*_*k.*min_ ≤ *f*_*k*_ ≤ *f*_*k*.max_ *k* = 1, …, *N*	Objective space (space of objective vectors defined by ranges for performance figures the surrogate is to cover)	*X* = [***l u***] ***l*** = [*l*_1_ …, *l*_*n*_]^*T*^ ***u*** = [*u*_1_ …, *u*_*n*_]^*T*^ *l*_*i*_ ≤ *x*_*i*_ ≤ *u*_*i*_ *i* = 1, …, *n*	Conventional parameter space delimited by lower and upper bounds ***l*** and ***u*** on circuit parameters
***f*** = [*f*_1_ … *f*_*N*_]^*T*^	Objective vector (set of performance figures)	***x*** = [*x*_1_ … *x*_*n*_]^*T*^	Parameters vector (set of circuit dimensions)

In order to clarify the matter, consider a microstrip coupler whose performance figures include the operational frequency *f*_0_ along with the power split ratio *K*_*P*_. If the aim is to improve the matching and port isolation at *f*_0_, and also to ensure the assumed power split, the possible formulation of the function *U* is

1 $$ \begin{aligned} U({\varvec{x}},{\varvec{f}}) & = U({\varvec{x}},[f_{0} \;K_{P} ]^{T} ) = \max \left\{ {|S_{11} ({\varvec{x}},f_{0} )|,|S_{41} ({\varvec{x}},f_{0} )|} \right\} \\ & \quad + \beta \left[ {(|S_{31} ({\varvec{x}},f_{0} )| - |S_{21} ({\varvec{x}},f_{0} )|) - K_{P} } \right]^{2} \\ \end{aligned} $$

Here, the goal is minimization of |*S*_11_| and |*S*_41_| at *f*_0_ (in a minimax sense), whereas the second term is used to assess the deviation between the actual and target power split at the center frequency.

The performance metric *U*(***x***,***f***) serves to determine the solution x^*^ that is optimum for a given objective vector ***f*** ∈ *F*. We will use notation ${\varvec{x}}^{*} = U_{F} ({\varvec{f}}) = \arg \mathop {\min }\limits_{{{\varvec{x}} \in X}} U({\varvec{x}},{\varvec{f}})$. It should be observed that the notation x^*^ = *U*_*F*_(***f***) = argmin{***x*** ∈ *X* : *U*(***x***,***f***)} constitutes a simplification with the underlying assumption that the solution to this minimization problem is unique. In practice, the uniqueness is not always guaranteed, although non-uniqueness is unlikely for the class of problems considered in the work, where design specifications are imposed on vector-valued system outputs, thereby making the optimization task heavily overdetermined. Nonetheless, possible non-uniqueness might be readily addressed by using regularization. The set comprising all optimal designs obtained for all ***f*** ∈ *F* is referred to as the optimum design manifold *M*_*F*_ = *U*_*F*_(*F*)^76^, and it constitutes an *N*-dimensional entity within the space *X*. From the design applications standpoint, it suffices to render the surrogate on the manifold *M*_*F*_ only, as such a model would be sufficient to represent all designs of satisfactory quality for any objective vector within *F*. However, spatial allocation of *M*_*F*_ is unknown, only individual vectors can be identified for specific objective vectors ***f***.

Recently, a modeling approach has been introduced^83^, where determination of the surrogate model domain relies on statistical methods. In^83^, the approximation of *M*_*F*_ has been performed with the use of randomly allocated trial points (observables) in the design space *X*, specifically, using information about the operating conditions at these points, obtained from EM-simulated system responses. Yet, as the random points are unlikely to be of good quality, the optimum design manifold is approximated using a regression model *s*_*r*_(***f***) of limited number of degrees of freedom^83^, which yields the trend functions established in the least-square sense. Subsequently, the metamodel domain is identified through appropriate orthogonal extension of the initial approximation^83^ in *X*_*S*_ using any approximation technique of choice, e.g., kriging^84^ or neural networks^85^. In this work, we advance over the technique presented in^83^ by enhancing it with dimensionality reduction mechanisms.

In the nested kriging, domain definition procedure based on pre-optimized reference designs, the acquisition cost of which was substantial, despite the fact that some methods for accelerating reference design acquisition have been available (e.g.,^86^). Here, instead, the initial step of defining the model domain is to render a set of trial points ***x***_*r*_^(*j*)^, *j* = 1, 2, …, in the original parameter space *X*, referred to as observables. The points are allocated within the respective ranges for all parameters, using uniform probability distribution. Each observable is associated with the EM evaluation of the circuit at the respective vector ***x***_*r*_^(*j*)^. This data is used to extract the corresponding performance figure ***f***_*r*_^(*j*)^.

**Figure 1:**
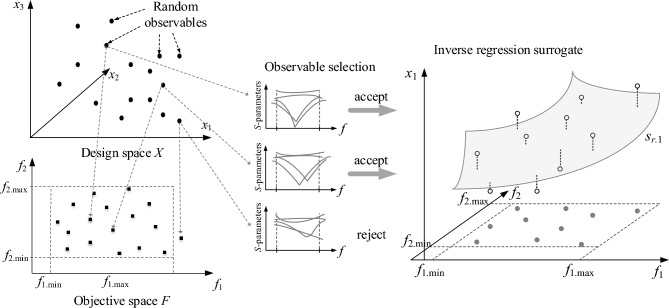
Generation of observables (left panel) for a microstrip coupler (three-dimensional design space *X*, two-dimensional objective space *F*); sample selection (middle panel): observables whose operating frequency and power split ratio fall into *F* are kept, the remaining samples are not taken into account; inverse model *s*_*r*_(⋅) construction utilizing the set of observables {***x***_*r*_^(*j*)^}_*j* = 1,…,*Nr*_, (right panel) for a single component of *s*_*r.j*_, representing parameter ***x***_1_ (marked as gray manifold).

Let us now go back to the microwave coupler example, where the objective space is two-dimensional and contains the intended operating frequency and target power split ratio. If the design is away from the optimum, the frequencies representing the minimum of matching |*S*_11_| and isolation |*S*_41_| characteristics may be severely misaligned, and the actual operating frequency may be taken as the average of the two values with the power split ratio calculated at this very frequency. If the extracted performance figure vector ***f***_*r*_^(*j*)^ is within the assumed space of design objectives *F*, the observable ***x***_*r*_^(*j*)^ is accepted; otherwise, it is rejected. Figure 1 graphically illustrates of the random sampling process described above.

The required number *N*_*r*_ of accepted samples is a control parameter of the modeling procedure, which is normally set to 50 or 100. This is sufficient to yield reasonable approximation of the manifold where the optimum designs reside for microwave circuits described by up to ten or so parameters. At the same time, the actual number of observables that need to be generated to obtain *N*_*r*_ accepted ones is typically 2*N*_*r*_ to 3*N*_*r*_, primarily depending on the size of the objective space *F* (i.e., the ranges of the operating conditions). 

Together with the vectors comprising objectives ***f***_*r*_^(*j*)^, the observables are also used to extract additional information pertaining to the quality of the design ***x***_*r*_^(*j*)^. This information is then encoded in the scalar coefficients *p*_*r*_^(*j*)^, *j* = 1, …, *N*_*r*_, defined in such a way that smaller coefficient values are assigned to better designs. This is to distinguish between designs that reside in the proximity to the optimal design manifold *M*_*F*_ (these will have a higher impact on the inverse model to be constructed therefrom), and others, being farther away from *M*_*F*_. In the consider example, where the aim was to improve impedance matching and port isolation of the component at the operating frequency, and also to enforce equal power split. Thus, the coefficient *p*_*r*_^(*j*)^ may be taken as the maximum of |*S*_11_| and |*S*_41_| at the assessed operating frequency, the latter being the first entry of the vector ***f***_*r*_^(*j*)^.

In^83^, the observable data is utilized to render the inverse regression surrogate *s*_*r*_: *F* → *X* approximating the set of optimum designs *M*_*F*_. Next, the image *s*_*r*_(*F*) is extended to become the domain of the final surrogate model. In this work, it will become the foundation for the spectral analysis, thereby leading to a dimensionality-reduced domain, as described in “Reduced-Dimensionality Model Domain. Construction of Final Surrogate” Section.

### Reduced-dimensionality model domain. Construction of final surrogate

The goal is to identify a domain that has a lower dimensionality with respect to that of the conventional design space *X*. The anticipated benefits are lower computational expenses associated with the setting up the surrogate, and improved scalability, i.e., more advantageous relationship between the training dataset cardinality and modeling error.

**Figure 2:**
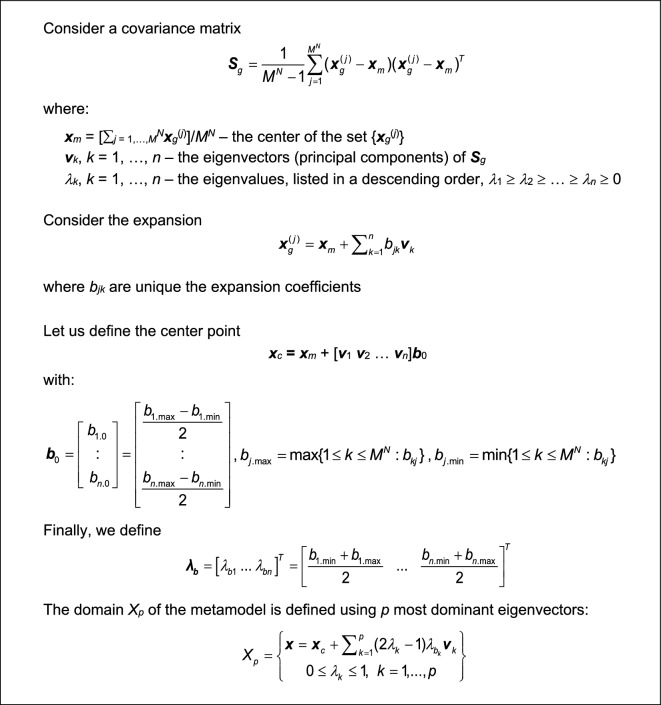
Procedure for identification of the dimensionally-reduced surrogate domain.

Let us define a rectangular grid *F*_*g*_ ⊂ *F*, as a set of all objective vectors ***f***_*g*_ = [*f*_*g.*1_ … *f*_*g.n*_]^*T*^ of the form

2 $$ {\varvec{f}}_{g} = \left[ \begin{gathered} f_{g.1} \\ \vdots \\ f_{g.N} \\ \end{gathered} \right] = \left[ \begin{gathered} f_{1.\min } + \left( {f_{1.\max } - f_{n.\min } } \right)\frac{{m_{1} }}{M - 1} \\ \vdots \\ f_{N.\min } + \left( {f_{N.\max } - f_{N.\min } } \right)\frac{{m_{N} }}{M - 1} \\ \end{gathered} \right] $$

where *m*_*k*_ ∈ {0, 1, …, *M* – 1}, *k* = 1, …, *N*. To put it another way, the grid *F*_*g*_ contains *M*^*N*^ vectors ***f***_*g*_^(*j*)^, *j* = 1, …, *M*^*N*^, uniformly distributed in *F*. The grid density *M* is a control parameter of the modeling procedure, typically set to *M* = 5, but its specific value is not critical. The purpose of *F*_*g*_ is to gather a collection of parameter vectors ***x***_*g*_^(*j*)^ = *s*_*r*_(***f***_*g*_^(*j*)^), *j* = 1, …, *M*^*N*^, which serves to approximate the optimum design manifold. In order to define dimensionality-reduced domain of the surrogate model, we perform principal component analysis^87^ of {***x***_*g*_^(*j*)^}_*j* = 1, …, *M *_^*N*^, as presented in Fig. 2.

Because the set *X*_*p*_ (see Fig. 2) is spanned by the most significant eigenvectors, it accounts for the majority of parameter variations within the set s_r_(F). In practice, it is sufficient to use the first few vectors because the eigenvalues are quickly decreasing. For all verification examples of “Validation Experiments and Benchmarking” Section, we use *p* = 3. Needless to say, maintaining low dimensionality of the domain permits radical reduction in the number of training data points required to build an accurate surrogate. Figure 2 illustrates the procedure for surrogate model domain rendition. The surrogate itself is identified utilizing kriging interpolation^88^, based on the training samples allocated in *X*_*p*_ using Latin Hypercube Sampling (LHS)^89^, the details on design of experiment strategy can be found in^87^.

### Proposed modeling procedure: complete workflow

This section puts together all components of the modeling procedure introduced here. Before presenting the workflow, we briefly summarize the input and control parameters. The input variables, provided by the user, are the following:

- Original parameter space *X*. This is a conventional, interval-like set, determined by the lower and upper bounds on design variables, represented as vectors ***l*** and ***u***, respectively;
- Objective space *F*. This is the fundamental entity, derived from the formulation of the design task for which the surrogate is intended to be applied. The values of *f*_*k*.min_ and *f*_*k.*max_, being lower and upper bounds for the figures of interest, are decided upon to determine the surrogate’s region of validity.

The control parameters of the modeling procedure are outlined in Table 2. Observe that we only have three parameters, all straightforward to set up. One of these is the cardinality of the training dataset, which may be fixed, or adjusted adaptively, e.g., to reach a specific value of the modeling error. For the latter, the LHS-based design of experiments should be replaced by an appropriate sequential sampling methodology (e.g.,^90^).

**Table 2:** Dimensionality-reduced domain-confined modeling: Control parameters.

Parameter	Description and recommendations
*N*_*r*_	Cardinality of the observable set
	Recommended value: from 50 to 100; the higher the design space dimensionality *n*, the larger *N*_*r*_ should be used
*P*	Dimensionality of the surrogate domain
	Recommended value: *p* = 3 (in order to ensure good model scalability as a function of the number of training samples; should take into account the eigenvalues *λ*_*k*_)
*N*_*B*_	Cardinality of the training data set (for surrogate model construction)
	Recommended values enabling to ensure relative RMS error of a few percent: from 200 to 500

**Figure 3:**
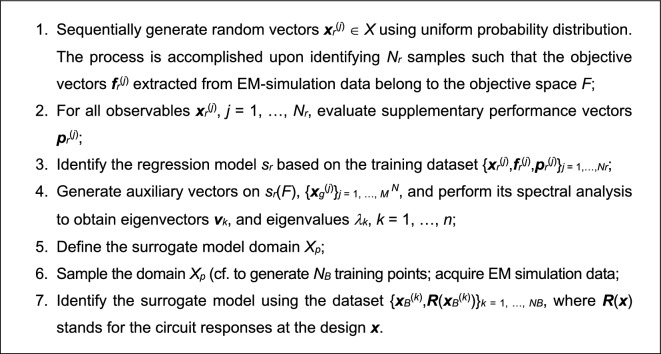
Operating flow of the introduced modeling procedure with domain confinement and dimensionality reduction.

Figure 3 summarizes the modeling process workflow. Although it is not explicitly mentioned in Fig. 3, the training data set {***x***_*B*_^(*j*)^} is supplemented by observables {***x***_*r*_^(*j*)^}, because this data is already available and allocated in the vicinity of the model domain. This will slightly improve the predictive power of the model, particularly for smaller training data sets. As a matter of fact, this improvement has been demonstrated in^83^. However, a similar effect is not expected to be as much pronounced within the introduced approach owing to a reduction of the domain dimensionality.

The proposed procedure exploits the performance-driven modeling paradigm^75^. As a result, it will exhibit similar characteristics as compared to conventional techniques operating in interval-like domains. These include:

- Improved immunity against the curse of dimensionality;
- A significant reduction of the domain volume translating into a smaller number of training data samples necessary to render high-accuracy surrogate;
- The ability to construct surrogates over extended ranges of both operational and geometry parameters.

Because of abandoning the concept of reference designs, similarly as in^83^, an additional advantage is the low initial cost of the model setup. Now, dimensionality reduction incorporated in this work leads to two additional benefits that are expected:

- Further improvement of the model predictive power without increasing the training data set size;
- Enhancement of the model scalability, that is, faster reduction of the modeling error as a function of increasing number of training samples.

All of these advantages are thoroughly demonstrated in the next section.

At this point, it should be mentioned that a possible limitation of the presented technique is that for excessively large parameter spaces, the observable set gathered in the pre-screening stage of the modeling process may be insufficient to cover the entire objective space. On the other hand, the likelihood of this to occur would be low if the original parameter space is established using engineering experience (as opposed to setting up excessively broad parameter ranges). At the same time, because normally weakly-nonlinear relationships between operating figures (e.g., center frequencies) and geometry/material parameters of the circuits, even if some parts of the objective space are left out in the sampling process, they may be recovered by evaluating the image of the regression model *s*_*r*_ for the objective space *F*. In other words, the regression model might extrapolate the missing parts, thereby providing the coverage of the entire optimum design manifold *M*_*F*_.

## Validation experiments and benchmarking

Here, we validate the modeling procedure introduced in “Reference-Design-Free Domain-Confined Modeling with Dimensionality Reduction” Section, and benchmark it against both conventional and performance-driven modeling methods. Numerical experiments are based on the following microstrip circuits, including two couplers: a rat-race and branch-line one, along with a dual-band power divider. The factors of interest are the modeling accuracy, computational cost of surrogate setup, as well as model scalability, i.e., dependence of the predictive power on the training data set cardinality. Furthermore, the surrogate constructed using the proposed approach is employed for circuit optimization to demonstrate its design utility.

### Verification circuits

Figure 5 shows the microwave components utilized in our verification experiments. Circuit I is a miniaturized rat-race coupler based on transmission line folding^91^. Circuit II is a compact branch-line coupler^92^, whereas the last circuit (Circuit III) is a dual-band power divider^93^. The information about the considered devices is gathered in Table 3.

**Table 3:** Verification circuits.

Parameter	Circuit structure		
	Circuit I^91^	Circuit II^92^	Circuit III^93^
Substrate	RO4003 (*ε*_*r*_ = 3.38, *h* = 0.76 mm)	*ε*_*r*_—operating parameter *h* = 0.76 mm	AD250 (*ε*_*r*_ = 2.5, *h* = 0.81 mm)
Design parameters^$^	***x*** = [*l*_1_* l*_2_* l*_3_* d w w*_1_]^*T*^	***x*** = [*g l*_1*r*_* l*_*a*_* l*_*b*_* w*_1_ *w*_2*r*_* w*_3*r*_* w*_4*r*_* w*_*a*_* w*_*b*_]^*T*^	***x*** = [*l*_1_ *l*_2_ *l*_3_ *l*_4_ *l*_5_ *s w*_2_]
Other parameters^$^	*d*_1_ = *d* +|*w − w*_1_|, *d* = 1.0, *w*_0_ = 1.7, and *l*_0_ = 15	*L* = 2*dL* + *L*_*s*_, *L*_*s*_ = 4*w*_1_ + 4* g* + s + *l*_*a*_ + *l*_*b*_, *W* = 2*dL* + *W*_*s*_, *W*_*s*_ = 4*w*_1 _+ 4* g* + *s* + 2*w*_*a*_, *l*_1_ = *l*_*b*_*l*_1*r*_, w_2_ = *w*_*a*_*w*_2*r*_, *w*_3_ = *w*_3*r*_*w*_*a*_, *w*_4_ = *w*_4*r*_*w*_*a*_	*w*_1_ = 2.2 mm, *g* = 1 mm
EM model	CST Microwave Studio	CST Microwave Studio	CST Microwave Studio
Figures of interest	Operating frequency *f*_0_ Power split ratio *K*_*P*_	Operating frequency *f*_0_ Substrate permittivity *ε*_*r*_	Lower band operating frequency *f*_1_ ratio *K*_*f*_ = *f*_2_/*f*_1_ between upper operating frequency *f*_2_ and *f*_1_
Objective space	GHz ≤ *f*_0_ ≤ 2.0 GHz –6.0 dB ≤ *K*_*P*_ ≤ 0 dB	1.0 GHz ≤ *f*_0_ ≤ 2.0 GHz 2.0 ≤ *ε*_*r*_ ≤ 5.0	2.5 GHz ≤ *f*_0_ ≤ 5.0 GHz 2.5 ≤ *ε*_*r*_ ≤ 4.5
Design optimality	Minimize matching and isolation at the target operating frequency *f*_0_ Maintain required power split ratio* K*_*P*_	Minimize matching and isolation at the target operating frequency *f*_0_ Maintain equal power split ratio	Minimize matching and isolation at both target frequencies *f*_1_ and *f*_2_ Maintain equal power division ratio
Conventional parameter space *X*	***l*** = [2.0 7.0 12.5 0.2 0.7 0.2]^*T*^, ***u*** = [4.5 12.5 22.0 0.65 1.5 0.9]^*T*^	***l*** = [0.4 0.43 5.9 7.7 0.68 0.28 0.1 0.1 2.0 0.2]^*T*^, ***u*** = [1.0 0.86 14.0 16.5 1.5 0.99 0.65 0.25 5.5 0.8]^*T*^	***l*** = [14.5 1.1 13.0 0.5 1.6 0.19 3.9]^*T*^, ***u*** = [37.0 16.6 35.0 15.0 5.6 1.5 5.8]^*T*^
Modelled characteristics	*S*-parameters: *S*_11_, *S*_21_, *S*_31_, *S*_41_,	*S*-parameters: *S*_11_, *S*_21_, *S*_31_, *S*_41_,	*S*-parameters: *S*_11_, *S*_21_, *S*_31_, *S*_41_,

^$^Dimensions in mm.

In all cases, the goal is to model the scattering parameters of the respective circuits as a function of frequency. The objective spaces are determined by the operating frequency and the target power split ratio (Circuit I), operating frequency and permitivity of the substrate used to fabricate the coupler (Circuit III), and target operating frequencies (Circuit III). In all cases, the relevant scattering characteristics are subject to the modeling process (e.g., *S*_11_, *S*_21_, *S*_31_, and *S*_41_ for the coupling structures). The modeling problems are demanding because of dimensionalities of the parameter spaces (up to ten), and also because of broad ranges of design variables. The latter can be measured using the average ratio of the upper to lower parameter bounds, which is about three for Circuits I and II, and as much as nine for Circuit III.

### Experimental setup

**Figure 4:**
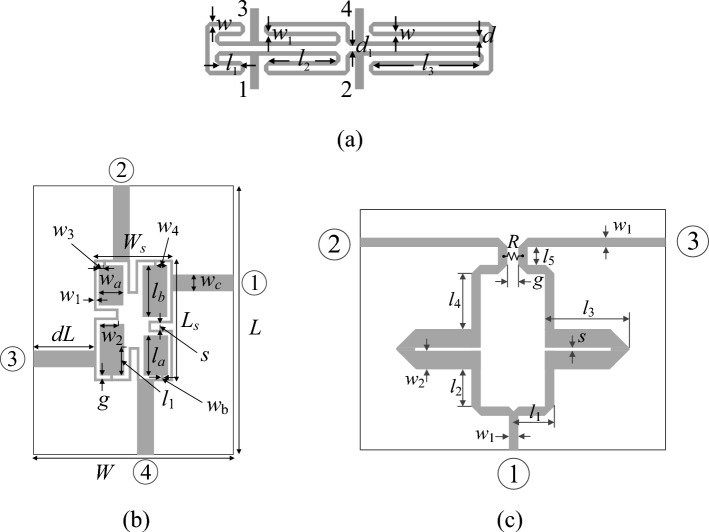
Microwave components used for verification of the introduced modeling framework: (**a**) Circuit I: rat-race coupler, (**b**) Circuit II: branch-line coupler, (**c**) Circuit III: dual-band power divider.

The proposed framework has been applied to construct surrogate models for all three circuits shown in Fig. 4. The control parameter *N*_*r*_ (the required number of accepted observables) is set to hundred in the case of Circuits I and II, whereas for Circuit III, it is fifty. The number of random points actually generated to reach *N*_*r*_ was 116, 226, and 78, for Circuit I, II, and III, respectively. The dimensionality of the design space is set to *p* = 3 (Circuits I and II), and *p* = 2 (Circuit III), which is because the last structure is a considerably more difficult as shown later on. Furthermore, the surrogate models were built for different training sets comprising the following numbers of samples: *N*_*B*_ = 50, 100, 200, 400, and 800. The purpose was to look into the scalability of the modeling error, i.e., its dependence on the number of training samples.

Table 4 outlines the benchmark methods employed in our comparative experiments. These include five state-of-the-art models established in the design space *X*, and also two performance-driven models rendered in confined domains, defined according to the particular modeling method. It should be reiterated that the paper addresses behavioral (data-driven) modeling, therefore, all benchmark techniques belong to this category. Furthermore, we do not take into account sequential sampling methods.

**Table 4:** Benchmark techniques.

Modeling technique	Domain	Comments
Kriging interpolation	Conventional (parameter space *X*)	Gaussian correlation function, second-order polynomial used as a trend function
Radial basis functions (RBF)	Conventional (parameter space *X*)	Gaussian correlation function, scaling coefficient determined through cross-validation
Artificial neural networks (ANN)	Conventional (parameter space *X*)	Feedforward network with two hidden layers, model training using backpropagation
Convolutional neural networks (CNN)	Conventional (parameter space *X*)	Model with 4 filters with the filter sizes of [64 128 256 512] trained with the ADAM algorithm
Ensemble learning	Conventional (parameter space *X*)	Least-squares boosting with 500 learning cycles, Learning rate optimized through Bayesian optimization
Nested kriging^76^	Confined domain *X*_*S*_	Thickness parameter *T* = 0.1, the following number of reference designs used: Circuit I (12 designs, acquisition cost 779 EM analyses) Circuit II (9 designs, acquisition cost 1014 EM analyses) Circuit III (9 designs, acquisition cost 923 EM analyses)
Reference-design-free modeling^83^	Confined domain *X*_*S*_	Thickness parameter *T* = 0.05 (Circuits I and II) and *T* = 0.025 (Circuit III), the following numbers of EM simulation used to identify *N*_*r*_ = 50 accepted observables: 116 (Circuit I) 226 (Circuit II) 78 (Circuit III)

The predictive power of the constructed metamodels has been assessed as a relative RMS error, defined as ||***R***_*s*_(***x***) − ***R***_*f*_(***x***)||/||***R***_*f*_(***x***)||, in which ***R***_*s*_ and ***R***_*f*_ are the frequency characteristics rendered by the model and full-wave simulations, respectively. The calculation of the error involves one hundred random testing samples, independent of the training ones.

### Results

**Figure 5:**
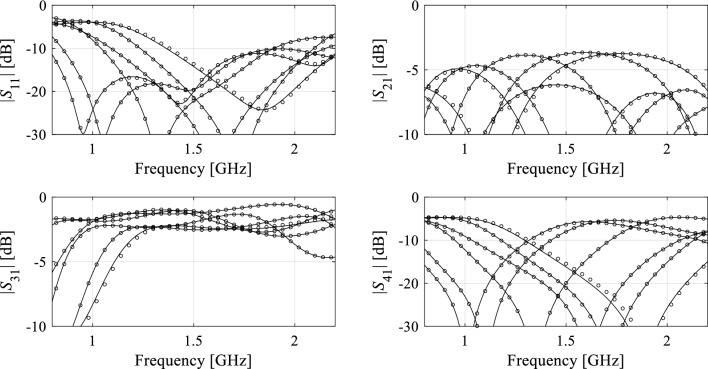
Circuit I: S-parameters for the representative test designs: full-wave simulated (—), and surrogate-predicted response (o). *N*_*B*_ = 400 training samples have been used to identify the model.

**Figure 6:**
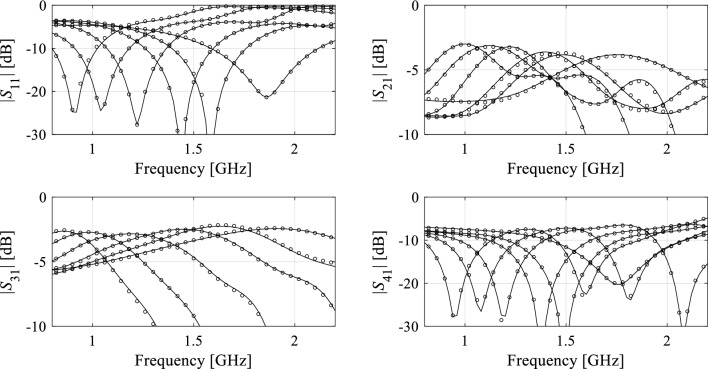
Circuit II: S-parameters for the representative test designs: full-wave simulated (—), and surrogate-predicted response (o). *N*_*B*_ = 400 training samples have been used to identify the model.

**Figure 7:**
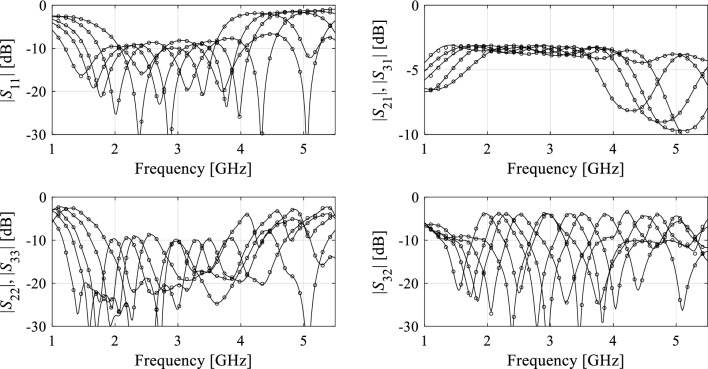
Circuit III: S-parameters for the representative test designs: full-wave simulated (—), and surrogate-predicted response (o). *N*_*B*_ = 400 training samples have been used to identify the model.

Tables 5, 6, and 7 show the results achieved for Circuit I through III. Meanwhile, Figs. 5, 6, and 7, present the juxtaposition of the model-predicted and EM-evaluated outputs of the respective structures, each for five selected test points. Based on the results, one can make the following observations:

- The surrogates’ predictive power constructed with the proposed approach is considerably improved w.r.t. conventional metamodels (kriging, RBF, ANN, CNN, Ensemble learning), all set up over unconfined design space *X*. Moreover, the predictive power of conventional surrogates is far from satisfactory even for the training sets of largest cardinalities (*N*_*B*_ = 400 and 800), which indicates that the considered modeling tasks are challenging;
- Our surrogate is also superior over the nested kriging framework. On the one hand, this is due to incorporating the observable data into the training set, which is especially pronounced for reduced-number training sets (*N*_*B*_ = 50 and 100). On the other hand, it is because of lower dimensionality of the surrogate domain (*p* = 3; in the case of Circuits I and II, whereas *p* = 2 has been used for Circuit III), which translates into a faster decrease of the modeling error for larger training sets (*N*_*B*_ = 200, 400, and 800). For the same reason, the decrease is much slower for the nested kriging.
- When compared to the reference-design-free method^83^, the proposed surrogate is slightly worse in terms of accuracy only for the smaller training data sets (*N*_*B*_ = 50). Although both techniques incorporate observable data into the training set, it is more beneficial for the model^83^ due to full dimensionality of its domain. For larger training sets, i.e., *N*_*B*_ ≥ 100, the predictive power of our surrogate is superior over^83^, primarily due to improved scalability.
- Reduced computational cost of constructing the model is another advantage of the proposed approach. As no reference designs are required, the expenses related to domain definition are dramatically lower than for the nested kriging framework^76^. The relative savings are as high as 80, 74, and 87% for *N*_*B*_ = 50, for Circuit I, II, and III, respectively. The savings for *N*_*B*_ = 800 are 42, 43, and 4% for the respective circuits. As indicated in “Reference-Design-Free Domain-Confined Modeling with Dimensionality Reduction” Section, the setup cost is the same for the presented technique and the modeling approach of^83^, as both methods utilize the same preliminary steps for domain rendition.

**Table 5:** Circuit I: Modeling results.

Modeling method	Number of training samples				
	50	100	200	400	800
Kriging					
Modeling error	25.7%	17.9%	13.5%	9.9%	8.0%
Model setup cost	50	100	200	400	800
RBF					
Modeling error	28.3%	19.1%	13.9%	10.3%	8.9%
Model setup cost	50	100	200	400	800
ANN					
Modeling error	18.2%	12.2%	8.0%	7.8%	6.5%
Model setup cost	50	100	200	400	800
CNN					
Modeling error	22.9%	12.7%	8.0%	5.5%	4.5%
Model setup cost	50	100	200	400	800
Ensemble learning					
Modeling error	32.7%	28.1%	25.0%	22.8%	19.1%
Model setup cost	50	100	200	400	800
Nested kriging^76^					
Modeling error	6.9%	5.7%	3.8%	3.5%	3.1%
Model setup cost^$^	829	879	979	1,179	1,579
No-reference-design modeling^83^					
Modeling error	4.8%	4.2%	3.3%	3.2%	2.6%
Model setup cost^#^	166	216	316	516	916
No-reference-design reduced- dimensionality modeling (this work)					
Modeling error	2.9%	2.6%	2.2%	1.5%	1.3%
Model setup cost^#^	166	216	316	516	916

^$^The cost includes acquisition of the reference designs, which is 779 EM simulations of the circuit.

^#^The cost includes generation of random observables, here, 116 simulations in total to yield *N*_*r*_ = 100 accepted samples.

**Table 6:** Circuit II: modeling results.

Modeling method	Number of training samples				
	50	100	200	400	800
Kriging					
Modeling error	52.3%	38.3%	31.0%	27.3%	23.3%
Model setup cost	50	100	200	400	800
RBF					
Modeling error	51.8%	40.5%	37.4%	32.8%	27.2%
Model setup cost	50	100	200	400	800
ANN					
Modeling error	29.9%	22.2%	15.2%	10.5%	9.8%
Model setup cost	50	100	200	400	800
CNN					
Modeling error	51.9%	39.9%	30.7%	19.7%	11.5%
Model setup cost	50	100	200	400	800
Ensemble learning					
Modeling error	53.1%	44.4%	41.6%	38.7%	33.3%
Model setup cost	50	100	200	400	800
Nested kriging^76^					
Modeling error	10.0%	7.4%	6.8%	5.1%	4.8%
Model setup cost^$^	1,064	1,114	1,214	1,414	1,814
No-reference-design modeling^83^					
Modeling error	7.6%	6.2%	4.7%	4.5%	3.4%
Model setup cost^#^	276	326	426	626	1,026
No-reference-design reduced- dimensionality modeling (this work)					
Modeling error	9.2%	5.6%	3.9%	2.8%	2.5%
Model setup cost^#^	276	326	426	626	1,026

^$^The cost includes acquisition of the reference designs, which is 1,014 EM simulations of the circuit.

^#^The cost includes generation of random observables, here, 226 simulations in total to yield *N*_*r*_ = 100 accepted samples.

**Table 7:** Circuit III: modeling results.

Modeling method	Number of training samples				
	50	100	200	400	800
Kriging					
Modeling error	63.6%	53.8%	45.2%	40.0%	35.1%
Model setup cost	50	100	200	400	800
RBF					
Modeling error	68.9%	55.2%	43.9%	40.8%	37.2%
Model setup cost	50	100	200	400	800
ANN					
Modeling error	36.7%	33.2%	24.6%	20.8%	20.3%
Model setup cost	50	100	200	400	800
CNN					
Modeling error	89.6%	44.7%	26.0%	17.8%	15.8%
Model setup cost	50	100	200	400	800
Ensemble learning					
Modeling error	47.8%	40.6%	38.1%	36.2%	33.6%
Model setup cost	50	100	200	400	800
Nested kriging^76^					
Modeling error	32.3%	19.2%	18.1%	15.2%	12.9%
Model setup cost^$^	973	1,023	1,123	1,323	1,723
No-reference-design modeling^83^					
Modeling error	23.7%	15.7%	10.8%	7.2%	6.1%
Model setup cost^#^	128	178	278	478	878
No-reference-design reduced- dimensionality modeling (this work)					
Modeling error	25.1%	15.4%	8.4%	3.6%	1.6%
Model setup cost^#^	128	178	278	478	878

^$^The cost includes acquisition of the reference designs, which is 923 EM simulations of the circuit.

^#^The cost includes generation of random observables, here, 78 simulations in total to yield *N*_*r*_ = 50 accepted samples.

### Design applications: circuit optimization

Surrogate models are constructed to facilitate EM-driven design procedures, primarily parametric optimization. In this section, we illustrate utilization of the models obtained using the proposed approach for optimization of Circuits I through III for various conditions. In particular, Circuit I has been optimized for a set of target operational frequencies and power split ratios, Circuit II for different operating frequencies and substrate permittivity, whereas Circuit III for different pairs of target frequencies corresponding to the lower and upper operating band. The notion of design optimality for the considered circuits has been explained in Table 3.

**Figure 8:**
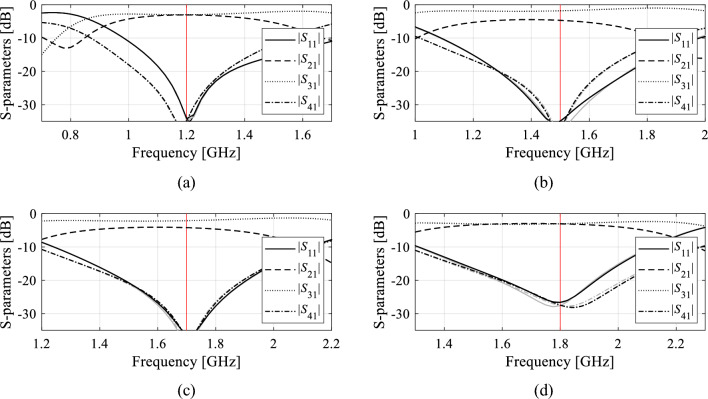
Circuit I (Fig. 4a). Surrogate-predicted *S*-parameters (gray lines) at the design rendered by optimizing the introduced domain-confined model with dimensionality reduction, built using *N*_*B*_ = 800 training samples. For comparison, EM-simulated design shown using black lines. The target operating frequencies are indicated by vertical lines: (**a**) *f*_0_ = 1.2 GHz, *K*_*P*_ = 0 dB, (**b**) *f*_0_ = 1.5 GHz, *K*_*P*_ = − 3 dB, (**c**) *f*_0_ = 1.7 GHz, *K*_*P*_ = − 2 dB, (**d**) *f*_0_ = 1.8 GHz, *K*_*P*_ = 0 dB.

**Figure 9:**
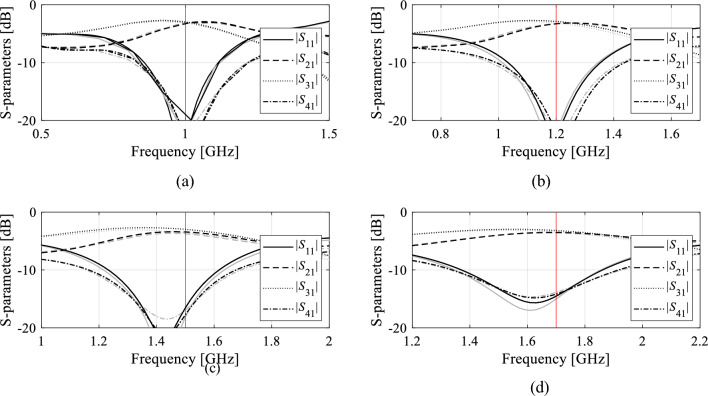
Circuit II (Fig. 4b). Surrogate-predicted *S*-parameters (gray lines) at the design rendered by optimizing the introduced domain-confined model with dimensionality reduction, built using *N*_*B*_ = 800 training samples. For comparison, EM-simulated design shown using black lines. The target operating frequencies are indicated by vertical lines: (**a**) *f*_*O*_ = 1.0 GHz, *ε*_*r*_ = 3.0, (**b**) *f*_*O*_ = 1.2 GHz, *ε*_*r*_ = 3.0, (**c**) *f*_*O*_ = 1.5 GHz, *ε*_*r*_ = 3.0, (**d**) *f*_*O*_ = 1.7 GHz, *ε*_*r*_ = 2.0.

**Figure 10:**
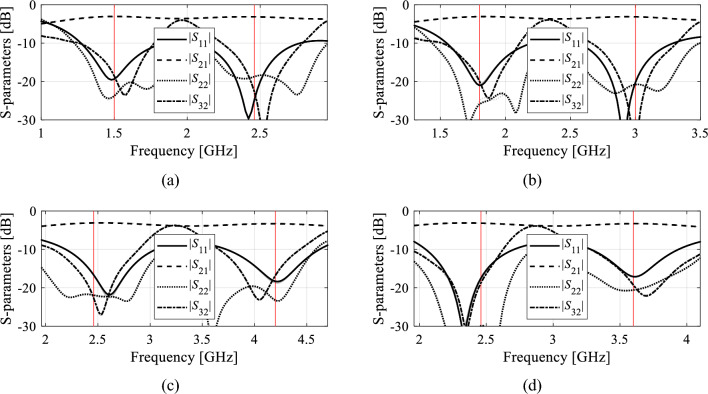
Circuit III (Fig. 4c). Surrogate-predicted *S*-parameters (gray lines) at the design rendered by optimizing the introduced domain-confined model with dimensionality reduction, built using *N*_*B*_ = 800 training samples. For comparison, EM-simulated design shown using black lines. The target operating frequencies are indicated by vertical lines: (**a**) *f*_l_ = 1.5 GHz, *K*_*f*_ = 1.63, (**b**) *f*_l_ = 1.8 GHz, *K*_*f*_ = 1.67, (**c**) *f*_l_ = 2.45 GHz, *K*_*f*_ = 1.71, (**d**) *f*_l_ = 2.45 GHz, *K*_*f*_ = 1.47.

The numerical data has been provided in Tables 8 through 10, which summarize the optimization goals, as well as geometry parameter values of the surrogate-optimized circuits. Circuit responses at the optimal designs are provided in Figs. 8, 9, and 10, for Circuit I, II, and III, respectively. Observe that the design objectives have been reached in all cases. Furthermore, the agreement between system responses predicted by the surrogates and those rendered through EM analysis is excellent. This indicates design utility of the models constructed using the proposed framework, in particular, their suitability to assist in designing the circuits within wide ranges of operating conditions without the necessity of further correction (Tables 9 and 10).

**Table 8:** Optimization results of Circuit I.

Target operating conditions		Geometry parameter values (mm)					
*f*_0_ (GHz)	*K*_*P*_ (dB)	*l*_1_	*l*_2_	*l*_3_	*d*	*w*	*w*_1_
1.2	0	3.56	8.62	18.77	0.42	0.94	0.76
1.5	− 3	3.43	9.45	15.45	0.39	1.00	0.53
1.7	− 2	3.45	9.23	13.96	0.35	0.87	0.54
1.8	0	3.82	8.67	13.52	0.33	0.78	0.73

**Table 9:** Optimization results of Circuit II.

Target operating conditions		Geometry parameter values									
*f*_0_ (GHz)	*ε*_*r*_	*g*	*l*_1*r*_	*l*_*a*_	*l*_*b*_	*w*_1_	*w*_2*r*_	*w*_3*r*_	*w*_4*r*_	*w*_*a*_	*w*_*b*_
1.0	3.0	0.87	0.61	9.71	15.31	1.14	0.66	0.37	0.19	4.61	0.51
1.2	3.0	0.79	0.61	8.81	13.07	1.18	0.63	0.37	0.19	3.95	0.52
1.5	3.0	0.72	0.61	8.72	11.51	1.24	0.60	0.37	0.18	3.45	0.53
1.7	2.0	0.72	0.63	9.73	12.02	1.30	0.57	0.36	0.18	3.49	0.55

**Table 10:** Optimization results of Circuit III.

Target operating conditions			Geometry parameter values (mm)						
*f*_1_ (GHz)	*K*_*f*_	*f*_2_ = *K*_*f *_*f*_1_ (GHz)	*l*_1_	*l*_2_	*l*_3_	*l*_4_	*l*_5_	*s*	*w*_2_
1.5	1.63	2.45	23.39	12.27	32.80	6.93	2.48	0.63	4.53
1.8	1.67	3.0	21.88	9.51	27.22	6.85	2.94	0.71	4.55
2.45	1.71	4.2	23.12	4.39	18.01	6.39	4.35	1.03	3.70
2.45	1.47	3.6	17.06	7.72	22.56	7.09	2.67	0.63	5.48

## Conclusion

This work introduced a novel technique for surrogate modeling of microwave passive components. Our methodology capitalizes on performance-driven modeling paradigm to define a low-volume domain utilizing a set of random observables and EM simulation data extracted therefrom, as well as the principal component analysis to enable a reduction of domain dimensionality. Incorporation of the aforementioned algorithmic tools permits a construction of reliable surrogate models valid over broad ranges of system parameters and its operating conditions, in particular, the operational frequency and material parameters (relative permittivity of the substrate the circuit is fabricated on). Furthermore, explicit dimensionality reduction significantly improves scalability properties of the surrogate, especially in terms of ensuring rapid increase in the model predictive power upon enlarging the training data set size. Extensive numerical experiments involving two microstrip couplers and a dual-band power divider demonstrate the improved predictive power of the proposed model as compared to several benchmark methods, both conventional and performance-driven. In particular, it allows achieving relative RMS error at the level of one to 3%, which is unattainable for the benchmark surrogates. At the same time, the computational cost of the model setup is significantly lower than the cost of the nested kriging framework, and it is the same as for the reference-design-free approach. This means that the accuracy improvement does not compromise the computational efficiency. Finally, a number of application case studies (circuit optimization), conducted for all three circuits, demonstrate practical usefulness of the proposed surrogate under a variety of design scenarios. A possible limitation of the presented technique is surrogate model scalability with respect to the number of performance figures. This is because the effective dimensionality of the domain is more or less equal to the number of those figures, which was two for all considered test cases. Increasing this number to three or four would considerably enlarge the effective domain dimensionality, which is expected to be detrimental to the scalability of the modelling error. On the other hand, scalability with respect to the number of the circuit parameters is expected to be much better due to the same reasons (low effective domain dimensionality). This was corroborated by comparing, e.g., modelling error for Circuit I (six parameters) and Circuit II (ten parameters), where the degradation of the predictive power of the surrogate was minor for the proposed technique, which was not the case for the majority of benchmark techniques.

The future work will include extension of the presented modelling technique to the class of physics-based surrogates such as space mapping. Another interesting topic for future studies would be to combine domain confinement methodology with sequential sampling techniques, e.g., Gaussian process regression with expected improvement as an infill criterion, which might lead to further improvements in terms of lowering the cost of graining data acquisition.

## Data Availability

The datasets generated during and/or analysed during the current study are available from the corresponding author on reasonable request.
